# Rethinking trophic niches: Speed and body mass colimit prey space of mammalian predators

**DOI:** 10.1002/ece3.6411

**Published:** 2020-06-28

**Authors:** Myriam R. Hirt, Marlee Tucker, Thomas Müller, Benjamin Rosenbaum, Ulrich Brose

**Affiliations:** ^1^ EcoNetLab German Centre for Integrative Biodiversity Research (iDiv) Halle‐Jena‐Leipzig Leipzig Germany; ^2^ Institute of Biodiversity Friedrich Schiller University Jena Jena Germany; ^3^ Senckenberg Biodiversity and Climate Research Centre (BiK‐F) Frankfurt Germany; ^4^ Department of Biological Sciences Goethe‐University Frankfurt Germany; ^5^ Department of Environmental Science Institute for Wetland and Water Research Radboud University Nijmegen the Netherlands

**Keywords:** ambushing, body mass ratio, food webs, group hunting, mega‐carnivores, movement, predator–prey interactions, prey range, pursuit predation

## Abstract

1. Realized trophic niches of predators are often characterized along a one‐dimensional range in predator–prey body mass ratios. This prey range is constrained by an “energy limit” and a “subdue limit” toward small and large prey, respectively. Besides these body mass ratios, maximum speed is an additional key component in most predator–prey interactions.
2. Here, we extend the concept of a one‐dimensional prey *range* to a two‐dimensional prey *space* by incorporating a hump‐shaped speed‐body mass relation. This new “speed limit” additionally constrains trophic niches of predators toward fast prey.
3. To test this concept of two‐dimensional prey spaces for different hunting strategies (pursuit, group, and ambush predation), we synthesized data on 63 terrestrial mammalian predator–prey interactions, their body masses, and maximum speeds.
4. We found that pursuit predators hunt smaller and slower prey, whereas group hunters focus on larger but mostly slower prey and ambushers are more flexible. Group hunters and ambushers have evolved different strategies to occupy a similar trophic niche that avoids competition with pursuit predators. Moreover, our concept suggests energetic optima of these hunting strategies along a body mass axis and thereby provides mechanistic explanations for why there are no small group hunters (referred to as “micro‐lions”) or mega‐carnivores (referred to as “mega‐cheetahs”).
5. Our results demonstrate that advancing the concept of prey ranges to prey spaces by adding the new dimension of speed will foster a new and mechanistic understanding of predator trophic niches and improve our predictions of predator–prey interactions, food web structure, and ecosystem functions.

## Graphical abstract

Trophic niches of predators are often characterized along a one‐dimensional range in predator–prey body mass ratios setting an "energy limit" and a "subdue limit" toward small and large prey, respectively. To this concept, we add a "speed limit" toward fast prey and show that predators of different hunting strategies separate along these two dimensions to avoid competition.

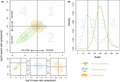

## 1 INTRODUCTION

Pursuing and capturing prey is as vital to the predator as it is to the prey to avoid capture. Thus, animals have evolved a variety of morphological traits and behavioral strategies that either maximize capture success or minimize predation risk (Cortez, 2011; Walker, Ghalambor, Griset, McKenney, & Reznick, 2005; Wilson et al., 2018). Predator avoidance strategies of prey have been intensely studied (Lima, 1998), but less attention has been paid to the behavior of the predator (Lima, 2002). More recent studies have focused on the interplay between predator and prey, and the determinants of predatory success in specific predator–prey pursuits, such as those between lion and zebra, or cheetah and impala (Wilson et al., 2015, 2018). Locomotor abilities such as speed and maneuverability are critical components in these interactions as well as body mass and hunting strategy (Bailey, Myatt, & Wilson, 2013; Bro‐Jørgensen, 2013; Caro, 2005; Wilson et al., 2018). While the determinants and success of specific predator–prey pursuits have thereby been analyzed explicitly, general determinants of predator–prey interactions and trophic niches are less explored. From the predator's perspective, this addresses the question: “which prey could possibly be captured and subdued?”

**FIGURE 1:**
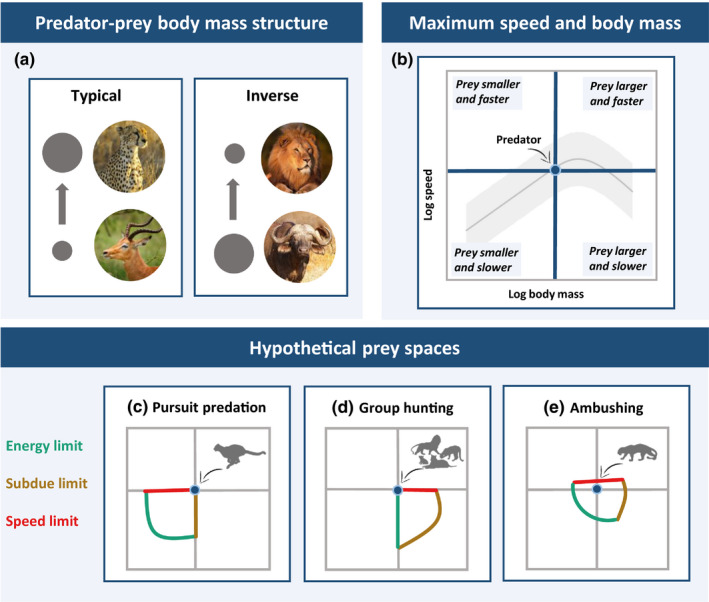
Concept of the prey space of different hunting strategies. (a) Typical and inverse body mass ratio of predators and their prey. (b) Scaling of maximum speed with body mass in terrestrial mammals. (c–e) Hypothesized prey space of pursuit predation (c), group hunting (d), and ambushing (e) set by the energetic, subdue, and speed limit. Panels c, d, and e have the same axes as panel b. Note that these limits are drawn as conceptual lines to illustrate our a priori expectations

Traditionally, this question on trophic niches was answered by placing predator and prey on a single body mass axis and setting lower and upper limits to the prey range predators can exploit (Brose, 2010; Portalier, Fussmann, Loreau, & Cherif, 2019; Schneider, Scheu, & Brose, 2012). Minimum prey size is determined by the “energetic limit”: The prey has to be large enough to meet the energy demands of the predator. This means that the energetic costs of hunting and attacking the prey should not exceed the energetic gain (Brose, 2010; Portalier et al., 2019). Maximum prey size is thereby determined by the “subdue limit”: The prey has to be small enough to be successfully subdued by the predator (Radloff & Du Toit, 2004). These limits differ depending on the movement mode of the predator and the dimensionality of the ecosystem (Brose et al., 2019; Pawar, Dell, Lin, Wieczynski, & Savage, 2019; Portalier et al., 2019). For typical terrestrial mammalian predators, these limits imply that prey body masses are between one and four orders of magnitude lower (Figure 1a, *typical predator–prey body mass structure,* Brose et al., 2006, Tucker & Rogers, 2014). However, there are exceptions to this pattern where predator–prey body mass ratios are inverted and predators are able to consume much larger prey (Figure 1a, *inverse predator–prey body mass structure*), such as lions (~200 kg) hunting African buffaloes (~650 kg). Lions are able to effectively subdue their larger prey because usually multiple group members are simultaneously attacking it.

**FIGURE 2:**
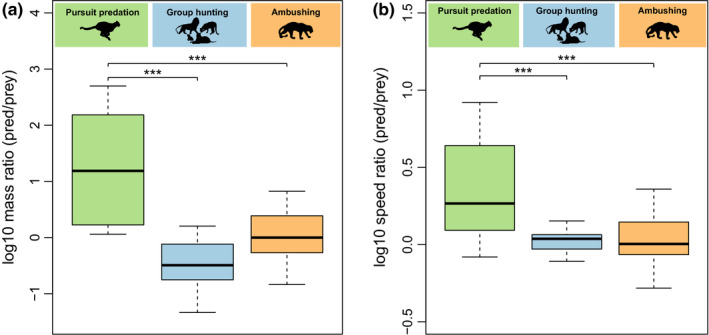
Prey range of predator–prey interactions of different hunting strategies. (a) Prey range of pursuit predation, group hunting and ambushing defined by body mass ratios between predator and prey. Body mass ratios of pursuit predation are significantly higher than those of group hunting or ambushing (*p* < 10^–5^). (b) Prey range of pursuit predation, group hunting, and ambushing defined by maximum‐speed ratios between predator and prey. Maximum speed ratio of pursuit predation is significantly higher than those of group hunting or ambushing (*p* < 10^–4^)

Besides body mass constraints, movement speed as well as acceleration, deceleration, and maneuverability play an important role during the hunting process (Wilson et al., 2018). Primarily, the predator needs to be fast enough to be able to catch its prey, which we refer to as the “speed limit.” Under the assumption that maximum speed follows a power–law relationship with body mass (Bejan & Marden, 2006; Hedenström, 2003; Peters, 1986), predators would generally not be able to catch prey that is much larger than they are, since it would always be faster. Therefore, the traditional concept of predator–prey body mass ranges cannot provide satisfying answers for the questions of (1) how the smaller predators manage to successfully catch their prey if the body mass structure of the interaction is inverse (Figure 1a) and (2) why there are no pursuit predators that are larger than mega‐herbivores (e.g., mega‐cheetahs). Therefore, a trophic niche concept that goes beyond simple predator–prey body mass ranges is necessary for a mechanistic understanding of interactions for both typical and inverse body mass structures (Figure 1a). Recent food web studies have revealed that the movement type (e.g., running, flying, swimming) of predator and prey is an important factor constraining their interactions (Brose et al., 2019; Jacob et al., 2011). Interestingly, the maximum speed of animals follows a hump‐shaped pattern with body mass (Garland, 1983; Hirt, Jetz, Rall, & Brose, 2017; Figure 1b), enabling medium‐sized predators to catch larger and therefore slower prey. This pattern adds a new dimension of speed to predator–prey interactions and generates a two‐dimensional “prey space” (across body mass and speed dimensions, Figure 1c–e) instead of a one‐dimensional “prey range” based on body mass. When analyzing this prey space, it is more informative to take into account differences in hunting strategies, as they might shift the limits in both dimensions (Bailey et al., 2013; Bro‐Jørgensen, 2013).

In this study, we develop a novel prey space concept that constrains the trophic niches of terrestrial mammalian predators with respect to body mass and maximum speed relative to their prey and assess systematic differences in this prey space according to three different hunting strategies: pursuit predation, group hunting, and ambushing (see Table 1 for definitions). We hypothesize that body mass and maximum speed should interactively determine the prey space of predators depending on their hunting strategy. Pursuit predators should not be able to consume prey that is larger (subdue limit) or faster in terms of maximum speed (speed limit) than they are (Figure 1c). Group hunters should have a high energetic limit, as prey needs to be shared between the group members. Therefore, they usually need to attack large prey to energetically sustain the whole group. This should be possible as the multiple agents involved in the attack enable them to subdue larger prey (higher subdue limit) (Bertram, 1979; Lamprecht, 1981; Packer & Ruttan 1988). Importantly, the novel hump‐shaped scaling model of maximum speed illustrates that they are also able to catch this larger (and therefore slower) prey (Figure 1d). Ambushers should be the most flexible in terms of these limits. By launching surprise attacks, they should be able to overwhelm larger and faster prey (Figure 1e). Consistent with prior predator–prey and food web studies (Brose, 2010; Petchey, Beckerman, Riede, & Warren, 2008; Schneider, Brose, Rall, & Guill, 2016), our concept focuses on the trophic dimension of the species’ niches. We illustrate our concept and test its hypotheses by compiling a database comprising data on body masses and maximum speeds of terrestrial mammalian predators and their dominant prey. Our empirical data is mostly limited by the availability of maximum‐speed data. Due to the further restriction to species pairs that are engaged in trophic interactions, our database comprises only 63 predator–prey links. While information on additional predator–prey interactions as well as body masses is available, extended and systematic empirical tests of our prey space concept would also require data on the maximum speed of predator and prey species. As some predators can switch between multiple hunting strategies (e.g., during different life stages), we characterize the hunting strategies for each of their links independently, which provides insights into the trophic niches of abstract hunting types. Finally, we shed light on the question of why there is no evolutionary tendency toward terrestrial mega‐carnivores with a body mass that exceeds that of the mega‐herbivores.

**TABLE 1:** Overview of the hunting strategies assigned to the predator–prey links. Precise descriptions of the hunting strategies pursuit predation, group hunting, and ambushing, and the predatory behaviors are included

Hunting strategy	Predatory behavior	Description
Pursuit predation	Pursuit	Active chase of prey over short or long distances. Sometimes includes stalking prior to a rapid chase
	Pounce‐and‐pursuit	
	Ambush‐and‐pursuit	
Group hunting	Pursuit over long distances	Cooperative hunters that actively chase their prey, usually over long distances.
Ambushing	Ambush	Either ambush and launch a surprise attack or capture prey by stalking followed by a short rush
	Stalk‐and‐ambush	

## 2 METHODS

### 2.1 Database assembly

We compiled data from the literature on the predominant prey species of 33 terrestrial carnivorous mammal species (see Table S1). Carnivores were defined as those species with diets compromising of at least 90% meat and only included species that consumed vertebrate prey (Kelt & van Vuren, 2001). Prey preference information was obtained using a literature search in Google Scholar using the terms “diet”, “prey preference” and each species’ name. The predominant prey species consumed was categorized as either (a) the preferred prey, calculated using the Jacob's Index, which standardizes the proportion of the total kills by the carnivore and the proportional availability of the prey species (Hayward & Kerley, 2005); or (b) the predominant prey species consumed by the carnivore based on diet analyses. For each species, we noted the top three to five prey species consumed depending on the number of species stated in the literature and their importance based on the percentage of the diet the prey represents. Diet information and preference information were included for both sexes and across populations. Although these predators have additional prey that they occasionally consume, we based our analyses only on the most frequently attacked or most preferred prey to characterize typical trophic niches. We then gathered data on body mass (Myhrvold et al., 2015) and maximum speed (Hirt, Jetz, et al., 2017) for these predator and prey species. However, maximum speed data were only available for 15 predator species and 419 of their prey species, which resulted in a database with 63 predator–prey links.

### 2.2 Hunting strategies

We assigned the hunting strategies pursuit predation, group hunting, and ambushing to these links. These hunting strategies comprise several predatory behaviors (Table 1). Pursuit predation summarizes “ambush‐and‐pursuit,” which includes an ambush part prior to the chase (e.g., a cheetah stalking a gazelle) and “pounce‐and‐pursuit,” which includes a pouncing part (e.g., a fox jumping to catch a mouse). Although pounce‐and‐pursuit is a predatory behavior between pursuit predation and ambushing, we assigned it to the category of pursuit predation because the pounce‐and‐pursuit predators in our study also show classical pursuit behavior (e.g., a fox preying on a mouse compared to a hare). Co‐operative hunting usually occurs in social animals living and hunting in groups (e.g., lions). They actively chase their prey mostly over longer distances. Ambushing means capturing prey by hiding or stalking in dense vegetation and launching a surprise attack (e.g., a cougar jumping on the back of a moose). As we defined the hunting strategy on the link level, a species can have multiple hunting strategies and predatory behaviors. For example, occasional group hunters such as wild dogs could occur as pursuit predators as well as group hunters or foxes may have pursuit as well as pounce‐and‐pursuit links.

### 2.3 Statistical analysis

We then analyzed the predator–prey links in a two‐dimensional space of body mass ratio (prey/predator) and maximum‐speed ratio (prey/predator; Figure 1). We used Bayesian modeling to estimate the prey space as the kernel of the predator–prey links by assuming a bivariate normal distribution of *x* = log(*mass ratio*) and *y* = log(*speed ratio)*. This normal distribution was fitted to the data under the constraint that the axis of largest variation (i.e., the first principal component) crosses the origin. As there is no unidirectional causal relationship between the two variables and because both have measurement errors of the same magnitude, we fitted a major axis regression with a fixed intercept of zero (the predator origin). We defined the prey space as the 90% confidence region of the found bivariate normal distribution, which takes the shape of an ellipsis (see Supplement for details of prey space estimation and Bayesian major axis regression). Moreover, we calculated the angles of the predator–prey links relative to the *y*‐axis in clockwise orientation (0°–360°) for each hunting strategy.

## 3 RESULTS

In this study, we have analyzed body masses in combination with maximum speeds of mammalian predators and their prey. In the following, we first illustrate the concept of prey *ranges* along these two axes. Predator body mass and maximum speed are expressed relative to the prey values, which yields prey ranges in terms of predator to prey body mass ratios (hereafter: mass ratio) and predator to prey maximum‐speed ratios (hereafter: speed ratio). For illustration purposes, we use the log10 values of these ratios in Figure 2. Subsequently, we extend this concept of prey ranges by combining these two axes creating two‐dimensional prey *spaces.* Finally, we test our hypotheses on differences in prey spaces according to the hunting type characterizing the predator–prey link (pursuit predation, group hunting, and ambushing).

The concept of prey ranges can be easily described using the cheetah (*Acinonyx jubatus*) as an example of a pursuit predator. Thomson's gazelle (*Eudorcas thomsonii*) is one of its prey species. In our data, the body masses of the cheetah and the gazelle are 65 kg and 25 kg, respectively, which yields a mass ratio of 2.6 (log10 mass ratio of 0.41, see Table S2), implying that the cheetah is 2.6 times larger than the gazelle. The impala (*Aepyceros melampus*) with 54 kg is the largest prey of the cheetah in our database implying a mass ratio of 1.19 (log10 = 0.08). Together, this yields a prey range in terms of mass ratios between 1.19 and 2.6 (log10 range of 0.08 to 0.41) for the cheetah. As these mass ratios are normalized to predator mass and thus dimensionless, they can be pooled for all pursuit predators resulting in a prey range of mass ratios between 1.16 (log10 = 0.06, spotted hyena, *Crocuta crocuta*, and impala, *Aepyceros melampus*) and 500 (log10 = 2.7; jungle cat, *Felis chaus*, hunting mice, *Mus musculus*). This implies that pursuit predators typically choose their prey within the range of mass ratios of approximately equally sized (here: ratio of 1.16) to 500 times smaller individuals (mass ratio of 500, Figure 2a). Similarly, we can use the mass ratios to characterize the prey range of group hunters (0.05 to 5.3, log10 range of −1.3 to 0.72) and ambushing predators (0.14 to 6.69, log10 range of −0.84 to 0.83), which are significantly lower compared to pursuit predators (ANOVA, *p* < 10^–5^, Figure 2a). This results in the general pattern that pursuit predators are substantially larger than their prey, whereas ambushers are equally sized to a bit smaller, and group hunters equally sized to substantially smaller than their prey (Figure 2a).

While analyses of prey ranges typically employ body mass ratios, they can also be carried out using ratios between predator and prey maximum speed. In our database, the maximum speed of the cheetah is 120 km/h, whereas the maximum speed of its prey ranges between 65 km/h (Thomson's gazelle, *Eudorcas thomsonii*) and 97 km/h (springbok, *Antidorcas marsupialis*), which yields speed ratios between 1.24 and 1.85 (log10 range of 0.09 to 0.27, see Table S2). When pooled for the groups of predator hunting strategies, characteristic patterns emerge: pursuit predators typically have a much higher maximum speed than their prey, whereas group hunters have a similar or slightly higher speed, and ambushers exhibit a wider variety of lower or higher maximum speeds than their prey. These data show that the speed ratio of pursuit predators is significantly higher than those of group hunters or ambushers (ANOVA, *p* < 10^–4^, Figure 2b).

**FIGURE 3:**
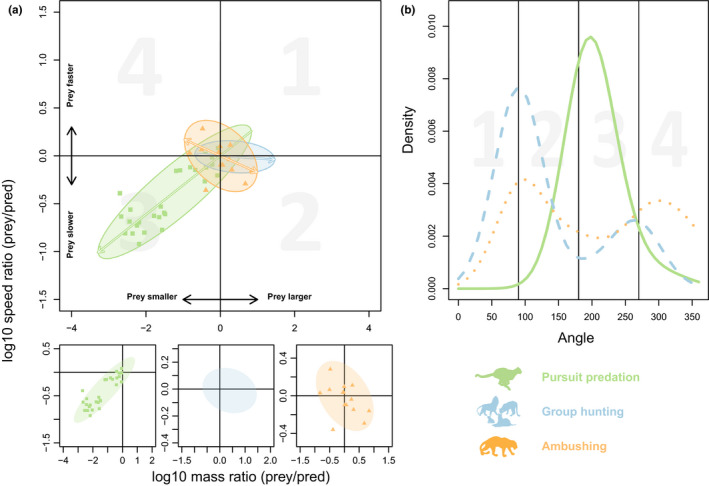
Prey space and niche differentiation of predator–prey links of different hunting strategy. (a) Prey spaces of the three different hunting strategies (pursuit predation, group hunting, ambushing) defined by body mass and maximum‐speed ratios of prey to predator. Small panels show prey spaces of pursuit predation, group hunting, and ambushing individually from left to right. Note that prey spaces and arrows are the same as in the large panel. Arrows indicate the main direction(s) of the predator–prey links. Slopes calculated by Bayesian major axis regressions: *b*_pursuit_ = 0.307 ± 0.018 (slope ± standard deviation, Bayesian posterior probability *P*(*b* > 0) = 1.00), *b*_group_ = −0.028 ± 0.034 (*P*(*b* < 0) = 0.805), *b*_ambusher_ = −0.185 ± 0.126 (*P*(b < 0) =0.942). (b) Niche differentiation by hunting strategy according to a combined effect of body mass and maximum speed. The angles refer to the angles of the feedings links in panel a

These analyses of the prey ranges in the dimensions of mass ratios (Figure 2a) and speed ratios (Figure 2b) demonstrate important differences in the niches of predators engaged in different hunting strategies. A more systematic and coherent analysis requires combining the two dimensions of the prey ranges described above to create a prey space (Figure 3a). For illustration purposes, mass and speed ratios are now expressed as prey to predator ratios. Any predator–prey interaction in this prey space is characterized by two values: the mass ratio as the *x*‐value and the speed ratio as the y‐value. For instance, the interaction between cheetah and Thomson's gazelle is placed at an x‐value of −0.41 (log10 prey to predator mass ratio) and a y‐value of −0.27 (log10 prey to predator speed ratio, see Table S2) in Figure 3a. Similar to the analyses of prey ranges, the data of all predator–prey interactions can be pooled in the same diagram as the values are normalized to predator body mass and predator maximum speed. Note that the predators of the interactions are thus positioned at the origin of the plot, as they have no difference in mass and speed to themselves. A feeding link between predator and prey in this prey space can be characterized by the angle of the connection between the origin (representing predator mass and maximum speed) and the data point (representing prey mass and maximum speed relative to the predator values). The quadrants of Figure 3a can thus be characterized as: (a) 0° to 90°—the prey is larger and faster (upper right), (b) 90 to 180°—the prey is larger and slower (lower right), (c) 180° to 270°—the prey is smaller and slower (lower left), and (d) 270° to 360°—the prey is smaller and faster than the predator (upper left).

The prey spaces were estimated using a bivariate normal distribution and Bayesian major axis regressions for each of the predator hunting strategies separately (Figure 3a). The difference between the prey spaces can be expressed by the differences in slopes and the distribution of data points across the four quadrants (Figure 3a). The prey space of pursuit predation is significantly different from the prey spaces of group hunting and ambushing as the slope (*b*) of the pursuit prey space is significantly higher than that of group hunting (*P*(*b*_pursuit_ > *b*_group_) = 1.00, Bayesian posterior probability) and ambushing (*P*(*b*_pursuit_ > *b*_ambush_) = 0.999). Moreover, all prey links of pursuit predators occupy the lower left quadrant (main direction of links towards quadrant 3, Figure 3a), indicating that they only hunt prey that is smaller and slower. Group hunters focus on prey that is larger and slower than they are (main direction of links towards quadrant 2, Figure 3a), but also have some prey links that are close to the origin but distributed across the other quadrants (prey of roughly similar size and speed). Ambushers mainly hunt equally sized or larger prey that can be slower or faster than they are (Figure 3a, small panel on the right). The difference between group hunting and ambushing prey spaces, however, is not significant (*P*(*b*_ambush_ < *b*_group_) = 0.900), indicating that they occupy similar trophic niches that are clearly separated from pursuit predators. This can also be seen in the subsequent analyzes of the angles of the feeding links and their distributions (Figure 3b). The angles are calculated as the angle in degrees of the predator–prey link in Figure 3a relative to the y‐axis and thus also reflect the main direction(s) of the prey space. There is a clear niche differentiation between pursuit predation and group hunting: while pursuit predations mainly focuses on smaller and slower prey (angles around 200°, Figure 3b green line), group hunting occupies the surrounding niches with prey that is mostly larger and slower (around 80°, Figure 3b, left maximum of the blue line) or slightly smaller and slower (around 260°, Figure 3b, right maximum of the blue line). Ambushers occupy similar niches as group hunters but are less distinct.

## 4 DISCUSSION

In this study, we have extended the classical concept of prey ranges by integrating the dimensions of prey body mass and maximum‐speed, which defines trophic niches in a two‐dimensional prey space and found that the prey spaces of ambushing and group hunting are significantly different from that of pursuit predation. Previously, trophic niches were typically expressed as ranges on body mass axes where the minimum prey sizes consumed by predators are caused by the decreasing energy content of prey individuals making attacks energetically inefficient (Schneider et al., 2012). Maximum prey sizes of predators are constrained by the increasing power that is necessary to subdue the prey (Radloff & Du Toit, 2004). In our novel concept of prey spaces, the trophic niches are not only constrained by (a) the energy limit towards smaller prey sizes and (b) the subdue limit towards larger prey sizes but also by (c) the speed limit towards prey of higher speed. Together with a hump‐shaped scaling of maximum speed with body mass (Hirt, Jetz, et al., 2017), this concept yields predictions on the shape of the trophic niches of predators with different hunting strategies (i.e., pursuit predation, group hunting, ambushing). We provided a preliminary test of these hypotheses using a new database combining body mass and maximum speed of various mammalian predators and their prey. Subsequently, we will first discuss the trophic niche differentiation according to the different hunting strategies before we use the three niche limitations to argue why and how these hunting strategies are constrained. Moreover, we show how our novel prey space concept provides a mechanistic understanding of how the energy and speed limits might prevent the existence of small‐bodied group hunters (micro‐lions) and larger pursuit predators (mega‐cheetahs).

The prey space of pursuit predation fits the concept of a classical predator–prey pair where the predator is larger than its prey (typical body mass ratio, Figure 1a) as well as faster to be able to successfully capture it in a one‐to‐one chase (Figure 2). However, there is an obvious gap within the group of pursuit predation between the categories of classical pursuit and ambush‐and‐pursuit predation on the one side, and pounce‐and‐pursuit‐predation on the other side (Figure 2a, pursuit prey space). In the first category, predator–prey links have lower body mass and speed ratios than in the other categories, implying that classical pursuit predators and their prey are more similar in their morphology. Classical evolutionary theory assumes an arms race between pursuit predator and prey, which drives evolution of high speed in both. In this case, athletic capabilities of predator and prey closely match up (Bro‐Jørgensen, 2013; Wilson et al., 2018). In contrast, pounce‐and‐pursuit predators have considerable higher body mass ratios and therefore higher speed ratios, which implies that they feed on much smaller and slower prey than other pursuit predators. This may be due to a much lower energetic demand of pounce‐and‐pursuit predation compared with a classical one‐to‐one‐chase, which compensates for the low energetic gain of much smaller prey. Despite the differences between these types of pursuit predation, current limitations in data availability on maximum speed hampered a separation of the two categories. Consistent with our initial hypothesis, pursuit predators are mainly constrained by the speed and subdue limits of the prey space, while the energy limit differs between the categories.

Prey spaces of group hunting and ambushing are significantly different from that of pursuit predation. Interestingly, despite having evolved different hunting strategies, they occupy a similar trophic niche that avoids competition with pursuit predation. Group hunting shows an inverse body mass ratio with the prey mostly being larger than the predator (Figures 1a and 2). The multiple agents in the group reduce problems of locating and subduing large prey (Bertram, 1979; Lamprecht, 1981; Packer & Ruttan, 1988) and improve the kill efficiency (Schaller, 1972; Caraco and Wolf, 1975). However, to be able to successfully catch large prey, the prey individual should be equally fast or slower than the predator. This is only possible because the hump‐shaped scaling of maximum speed with body mass opens up a niche of larger and slower prey at intermediate to high body masses (Figure 1b). Thus, cooperative hunting is a good strategy for medium‐sized mammals to avoid competition within the prey space occupied by pursuit predators. However, group hunters are also able to catch prey that is slightly faster (Figure 2). This is probably due to differing individual hunting tactics within the group, which relax the speed pressure compared to a one‐to‐one chase. These hunting tactics include predator individuals circling prey and others waiting for the prey to capture it while trying to escape (Bailey et al., 2013; Stander, 1992). The hunting process also often includes an ambushing part where some group members hide while others chase the prey towards them (Bailey et al., 2013). Hunting in a group thus allows a shift in the subdue limit to large prey individuals and relaxes the speed limit, but it comes at the cost of higher energy limits as the prey is shared within the group.

Ambushing mainly focuses on equally sized to larger prey that can be slower as well as faster. They are able to subdue this larger prey because they launch surprise attacks like, for example, a cougar stalking a moose, leaping onto its back, and killing it by breaking its neck (Bartnick, Van Deelen, Quigley, & Craighead, 2013; Husseman et al., 2003). This tactic also enables them to catch faster prey. Thus, they are less restricted by the speed limit than the other hunting strategies, which come at the cost of other constraints (see detailed discussion below).

Our analysis shows that group hunting and ambushing open up new niches with prey that is not available to classical pursuit predation. However, the hunting strategies are not equally distributed among mammalian predators. The vast majority of carnivores are solitary hunters (80%–95%, Lührs & Dammhahn, 2010) either as pursuit predators (e.g., cheetahs, commonly foxes or lynx) or ambushers (e.g., tigers, jaguars, cougars, and leopards). Only few terrestrial mammalian predators are exclusively group hunters to the extent that all group members hunt simultaneously. This behavior is only found in lions (Packer, Scheel, & Pusey, 2015; Stander, 1992), dogs (Creel & Creel, 1995), bush dogs, dingoes, and wolves (Mech & Boitani, 2003). Many other social mammals hunt occasionally in groups such as hyenas, which also scavenge from conspecifics (Kruuk, 1972; Packer & Ruttan 1988), chimpanzees (Boesch, 1994), and many canid species (Krofel, 2008; Muntz & Patterson, 2004; Sillero‐Zubiri & Gottelli, 1995). Moreover, cooperative hunting is a more general phenomenon that also occurs among marine predators such a cetaceans and invertebrates such as ants. This poses the question of why group hunting is not more common among mammalian predators. Group hunting has some obvious disadvantages compared to pursuit predation: group hunters have a lower prey encounter probability and a smaller search area compared to pursuit predators (Fryxell, Mosser, Sinclair, & Packer, 2007; Scheel, 1993). For instance, the total search area of a lion pride consisting of five individuals is similar to the search area of a single lion. If all five individuals were solitary predators, their combined search areas and the resulting prey encounter rates would thus be about fivefold larger. Moreover, the prey needs to be shared between the group members. Thus, cooperative hunting can only evolve when the per capita rate of food intake within a hunting group is higher than that of a solitary individual (Packer & Ruttan, 1988). In addition, evolving sociality is a prerequisite of cooperative hunting and highly costly, though it can support and strengthen other forms of cooperation and bonds within groups. Being able to exploit the niche of larger prey, therefore, cannot completely counteract the costs of cooperative hunting such as the possible inadequacy of food distribution among group members and lower prey availability (Creel, 1997).

Ambushers are solitary hunters; however, they have even lower encounter rates with prey and a smaller search area than group hunters or pursuit predators (Crawley, 2009; Scharf, Nulman, Ovadia, & Bouskila, 2015; Werner & Anholt, 1993). Their ambush strategy requires dense vegetation to hide and stalk or wait for prey to come by (Beier, Choate, & Barrett, 1995). Their encounter rate therefore depends more on the activity of the prey than on their own mobility. Consequently, they invest little energy in searching but much time in waiting for prey (Crawley, 2009). This limitation in prey availability makes them less effective in foraging despite their prey space being larger than that of group hunters and more flexible than that of pursuit predators.

Pursuit predators have the largest search area and therefore a higher chance of encountering prey as they hunt individually while moving in open space. Energetically, however, classical pursuit predation is the most costly hunting strategy, as predators need to chase their prey with high speed. Therefore, they need to morphologically evolve high‐speed capacities and, additionally, a high maneuverability as well as quick acceleration and deceleration capacities (Wilson et al., 2013, 2015). While these aspects are of lower importance to group hunters, endurance speed plays a crucial role in their hunting tactic. For example, wolves often exhaust their prey over long distances (Bailey et al., 2013). Moreover, prey react differently to the appearance of predators. Smaller prey mostly tend to flee, while larger prey often stands in a defensive formation (Creel & Creel, 1995). The predators then attack from several directions and—if the prey starts to flee—a full‐speed chase begins. Thus, both endurance speed and maximum speed are important for the hunting success of group hunters. Furthermore, group hunters as well as ambushers need to evolve morphological features to be able to successfully kill much larger prey, such as massive jaws and long canines to bite the preys’ throat as found in lions or tigers. While group hunters have the additional advantage of a higher number of predator attacks on the prey by the different group members, ambushers mostly have a powerful build that enables them to bring down large prey.

**FIGURE 4:**
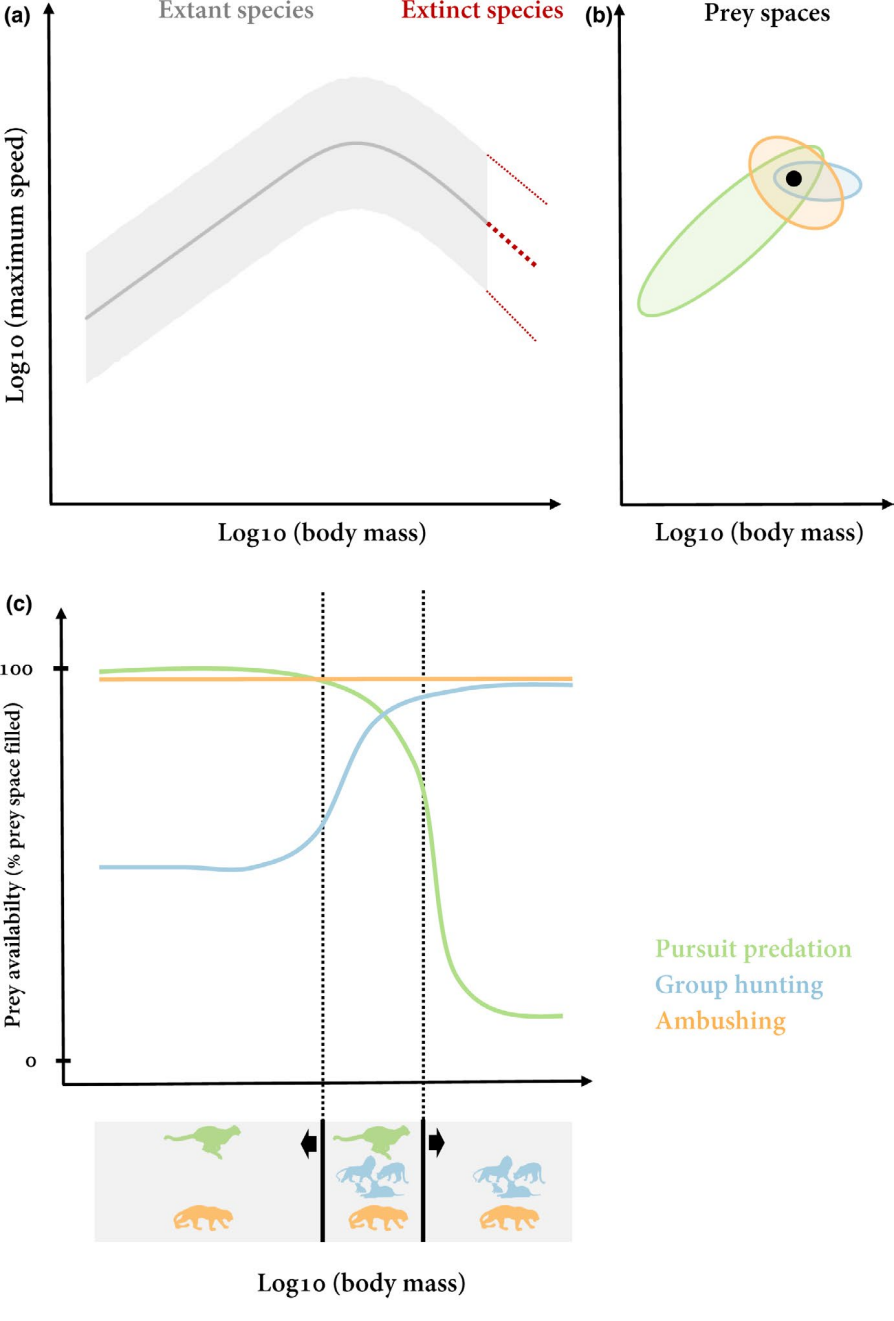
Hypothetical energetic optima of hunting strategies along the body mass axis. Based on the hump‐shaped scaling of maximum speed (a) and the prey spaces of the different hunting strategies (pursuit predator, group hunter, ambusher) (b), different energetic optima for each hunting strategy emerge (c). Prey availability is defined as the percentage of prey space that is filled with available prey species. Energetic optima are based on this prey availability combined with additional parameters such as encounter rates, attack success, or energy content of prey

Due to these morphological adaptations, predators should theoretically be able to develop each of the hunting strategies independent of their body mass. However, the hump‐shaped scaling of maximum speed with body mass (Figure 4a) results in each of the hunting strategies having a specific energetic optimum of its prey space (Figure 4b) along the body mass axis (Figure 4c). At lower predator body masses (below the hump), pursuit predation is energetically most effective as all of the prey space is filled (Figure 4c). Although the group hunting prey space is partially filled in this area, pursuit predation is the less costly hunting strategy and is therefore likely to be preferred. At intermediate body masses (at the hump), a predator can either evolve morphological adaptations for high‐speed movements to optimize its pursuit prey space or hunt cooperatively for larger prey. With increasing body masses above the hump, the pursuit prey space becomes continuously less filled due to limited availability of smaller and slower prey (Figure 4c). Hence, group hunting is energetically more reasonable within this body mass range (Figure 4c).

This general distribution of prey spaces might also explain why small group hunting mammals (micro‐lions) or large pursuit predators (mega‐cheetahs) are energetically unlikely to exist. For small social‐living carnivorous mammals, such as mongooses or meerkats, pursuit predation is energetically more reasonable despite their ability to cooperate in a group, because larger and slower prey are less available, and their prey space as group hunters would be less filled. Larger social animals, however, are able to develop cooperative hunting strategies, as the hump‐shaped scaling of maximum speed makes larger and slower prey available. Predators with larger body masses need to be either group hunters or ambushers, which are mostly smaller than their prey. Alternative theory on maximum body sizes of mammalian carnivores is based on constraints by the increased energy expenditure and intake linked to pursuing and consuming large prey, which should limit maximum carnivore size to approximately one ton (Carbone, Teacher, & Rowcliffe, 2007). While this limit applies to maximum sizes of terrestrial mammalian carnivores, extinct carnivorous reptiles had body sizes far beyond this limit of one ton (Burness, Diamond, & Flannery, 2001). The hump‐shaped scaling of maximum speed with body mass applies to all taxonomic groups including reptiles, indicating that the same constraints on prey spaces should be in effect. In past ecosystems, the largest herbivorous dinosaurs weighed approximately 70–80 tons (Mazzetta, Christiansen, & Fariña, 2004; Sellers, Margetts, Coria, & Manning, 2013) with maximum speeds of only 12 to 26 km/h, whereas predatory dinosaurs reached body masses of just up to 10 tons with maximum speeds of 27 to 55 km/h (Hirt, Jetz, et al., 2017; Sellers & Manning, 2007; Thulborn, 1982). It is generally accepted that many predatory dinosaurs hunted prey as large as or larger than themselves (Farlow & Holtz, 2002; Thomas & Farlow, 1997). Our prey space concept suggests that they should have either hunted in groups or ambushed their prey. For some dinosaur species, there is anecdotal evidence for this (Farlow, 1976; Mudroch et al., 2011; Ostrom, 1969; Xing et al., 2012), which provides additional support for our theory.

Here, we only extended trophic niches from one to two dimensions by including body mass and maximum speed in our concept, which are likely to be the key drivers. The speed dimension could further be complemented by endurance speed, which also plays a crucial role during the hunting process. Moreover, there are certainly more dimensions that could be added to this concept, which could either be direct species‐related effects (e.g., maneuverability (Wilson et al., 2018), diurnal versus. nocturnal activity (Emmons, 1987; Jaksić, 1982)), habitat domains, or indirect effects (e.g., habitat structure (Cresswell, Lind, & Quinn, 2010; Laundré, Hernández, & Ripple, 2010; Schmitz, Miller, Trainor, & Abrahms, 2017)). This is consistent with general analyses across ecosystems showing that trophic niches in complex food webs can be characterized by up to eight dimensions (Eklöf et al., 2013). The additional dimensions will help further explain or even shift the energetic optima of prey spaces along the body mass axis. For instance, our theory explains why pursuit hunters with high body mass ratios can probably not exist above the speed‐mass hump, as their prey spaces are not filled (Figure 4a). However, decreased maneuverability and acceleration/deceleration capacities impede a classical arms race between predator and prey and could therefore prevent the existence of pursuit predators with low body mass ratios, whose prey space would still be partially filled. Thus, our theory combined with additional dimensions of trophic niches suggests that large predators should be either group hunters or ambushers instead of pursuit predators.

Overall, empirical tests and natural patterns of predator–prey links support our mechanistic concept of extending the two body mass limits (“energy limit” and “subdue limit”) by a “speed limit” to define predator trophic niches in a two‐dimensional prey space across mammalian hunting strategies. Certainly, animals can at least partially overcome the constraints of body mass and speed on hunting success in many ways. In particular, learning abilities can create novel hunting techniques that could also be included as explanatory variables. However, our empirical tests were limited by the current lack of maximum speed data. The 33 predators of our database had 119 prey that they frequently consume yielding a total of 171 predator–prey links, but maximum speed data for predator and prey species were available for only 63 of these links. Our analysis of ~ 37% of the dominant interactions of these 33 predators should thus be interpreted as foundational yet preliminary support of our prey space concept. Further developments of this concept are dependent on more systematic assessments of maximum speed of predators and their prey, which will facilitate a more quantitative test of our niche‐space concept. These assessments could also include potential differences in maximum speeds in relation to different habitat types. Future extensions of our approach could also be generalized across predators of other phylogenetic groups (e.g., reptiles and invertebrates) and other ecosystem types (e.g., marine and freshwater). Here, studying the speed constraints on interactions between invertebrates could be facilitated by laboratory methods such as camera tracking (Hirt, Lauermann, Brose, Noldus, & Dell, 2017). As invertebrates are generally smaller than the threshold body mass of the maximum‐speed hump, it will be particularly interesting to include other movement modes (e.g., swimming and flying), as links between species of differing movement modes will yield “speed limits” that are entirely independent of body mass. Moreover, we anticipate that our prey space concept should be integrated with precise empirical assessments of the maximum speed and body mass of the predator and prey individuals that interact (Wilson et al., 2018), which will yield an unprecedented theory‐driven understanding of natural trophic niches.

Advancing the concept of prey ranges to prey spaces certainly deepens our mechanistic understanding of realized trophic niches in the wild. This is particularly important for predicting food web structures and the impacts of extinctions on predator–prey relationships. So far, body mass ratios helped comprehend the niche structure of natural communities (Brose et al., 2019; Woodward et al., 2005) and enabled predictions of food web structure by applying allometric diet breadth (Petchey et al., 2008) or allometric niche models (Schneider et al., 2016). This approach also successfully predicted effects of species loss on secondary extinctions and ecosystem functions (Brose et al., 2017; Schneider et al., 2012). The new concept of prey spaces illustrated here calls for a similar extension of one‐dimensional to two‐dimensional trophic niche models to improve our understanding of natural communities and better predict the consequences of extinctions for predator–prey interactions, food web structure, and ultimately ecosystem functions.

## CONFLICT OF INTEREST

None declared.

## AUTHOR CONTRIBUTION

**Myriam Rebecca Hirt:** Conceptualization (lead); Data curation (equal); Formal analysis (lead); Visualization (lead); Writing‐original draft (lead); Writing‐review & editing (lead). **Marlee Tucker:** Conceptualization (supporting); Data curation (equal); Writing‐original draft (supporting); Writing‐review & editing (supporting). **Thomas Müller:** Conceptualization (supporting); Writing‐original draft (supporting); Writing‐review & editing (supporting). **Benjamin Rosenbaum:** Formal analysis (supporting); Writing‐original draft (supporting); Writing‐review & editing (supporting). **Ulrich Brose:** Conceptualization (supporting); Formal analysis (supporting); Supervision (lead); Writing‐original draft (supporting); Writing‐review & editing (supporting). 

## Supporting information

Supplementary Material

## Data Availability

The full data set is available in the Supplement.
