# Supplementary material for: Rethinking trophic niches: Speed and body mass colimit prey space of mammalian predators
Source: Ecol Evol. 2020 Jun 28;10(14):7094–105. doi: 10.1002/ece3.6411 (PMC7391329; doi:10.1002/ece3.6411)
Supplement: Supplementary file 1 — Supplementary Material [file ECE3-10-7094-s001.pdf]

Supplementary Information for "Rethinking trophic niches: speed and body mass co-limit prey space of mammalian predators"

Hirt, M. R., T. Müller, B. Rosenbaum, M. Tucker, and U. Brose

Estimation of prey space

The prey space is defined by the 90% confidence region of a bivariate normal distribution, with the constraint that its first principal component (the axis of largest variation) crosses the origin. It is computed as follows.

The first principal component P_1 is determined by a major axis regression with fixed intercept zero (see below). Using its slope b, the principal component reads $P_1 = \binom{1}{b}$. The second principal component is orthogonal to the first one and therefore reads $P_2 = \binom{1}{-1/b}$ (such that $P_1^T P_2 = 0$). After normalization $P_1^{norm} = P_1/\|P_1\|$ and $P_2^{norm} = P_2/\|P_2\|$ ($\|\cdot\|$ denoting the Euclidean norm), the orthogonal 2x2 matrix of principal components is given by $P = [P_1^{norm}, P_2^{norm}]$.

Let $X = [x^1, \dots, x^n]$ be a 2xn matrix of n observations $x^i = \binom{x_1^i}{x_2^i}$. We perform a change of basis using the new basis P and transform the data $\tilde{X} = P^T X$. In this new space, the principal components are defined by the identity matrix $I = \begin{bmatrix} 1 & 0 \\ 0 & 1 \end{bmatrix}$, i.e. the data $\tilde{X}$ are uncorrelated. We fit an uncorrelated bivariate normal distribution to this transformed data $\tilde{X}$ by calculating its empirical mean vector $\mu_1 = \frac{1}{n}\sum_{i=1}^{n} \tilde{x}_1^i$, $\mu_2 = 0$ (to ensure that the mean vector is located on the first principal component) and empirical variances $\sigma_1^2 = \frac{1}{n-1}\sum_{i=1}^{n}\left(\tilde{x}_1^i - \mu_1\right)^2$, $\sigma_2^2 = \frac{1}{n-1}\sum_{i=1}^{n}\left(\tilde{x}_2^i - 0\right)^2$.

Using the mean vector $\mu = \binom{\mu_1}{\mu_2}$ and the covariance matrix $\Sigma = \begin{bmatrix} \sigma_1^2 & 0 \\ 0 & \sigma_2^2 \end{bmatrix}$, we determine the boundary $\tilde{E}$ (still in the transformed space) of the normal distribution's 90% confidence region with the R-package "ellipse". The prey space E for the original data X is calculated by performing a change of basis back to the original space $E = P\tilde{E}$.

Bayesian major axis regression

We use Bayesian methods for parameter estimation, i.e. we compute the posterior distribution of the parameters θ observing the data

$$P(\theta|data) = P(data|\theta) * P(\theta) / P(data),$$

where $P(data|\theta)$ denotes the likelihood function, $P(\theta)$ is the parameters' prior distribution and P(data) is a constant. Samples from the posterior distribution were drawn using Hamiltonian Monte Carlo sampling in *Stan*, accessed via the *RStan package* (Stan Development Team 2017).

For the Bayesian major axis regression, intercept *a* and slope *b* are sought, which minimize the sum of squared orthogonal distances of the observations (x,y) to the regression line (Warton et al. 2006). For any observation, the nearest point on the line is given by $x^*=(y+x/b-a)/(b+1/b)$ and $y^*=a+bx^*$ (orthogonal projection). The residuals are the Euclidean distances $d((x,y),(x^*,y^*))$ between the observations and their projections on the regression line and are modelled normally distributed with zero mean and standard deviation σ, which defines the likelihood function.

We fitted a model with a fixed intercept $a=0$ for all three groups of data (pursuit predator, group hunter, ambusher). Each group was fitted with its own slope b_i and standard deviation σ_i ($i=1,2,3$). We used vague priors for the slopes (normal distribution with mean 0 and standard deviation 10.0). For the residuals' standard deviations, a positive half-Cauchy prior (location 0.0, scale 2.0) was chosen (Gelman and Hill 2007). This Bayesian approach allows direct inference on all parameters and their combinations. I.e., from the samples of the posterior distribution, we can directly compute statistics on differences in slopes (b_i-b_j). We tested for significant differences by calculating posterior probabilities $P(b_i>b_j)$.

Clustering of predator-prey links into groups

We tested whether our a-priori categorization of the links into predation types according to the classification in Table 1 of the main manuscript has been driving our conclusions. We have thus used the kmeans clustering by the Hartigan–Wong algorithm (stats package of R version 3.5.3) to categorize the 63 predator-prey links of Supplementary Table 1 into three groups. We used the angle (angles of the predator-prey links relative to the y-axis in clockwise orientation (0°-360°) and the distance of the data point to the origin (square root of the sum over the squared mass ratios and the squared speed ratios) as independent variables.

The clustering yielded three groups of predator-prey links (Supplementary Fig. 1). Despite some differences in the classification of individual links (mostly data points close to the origin), the prey spaces grouped are consistent with those predicted using our a-priori classification in the main manuscript (see Fig. 3a of the main manuscript). In this vein, group 1 of Supplementary Fig. 1 (green data points) resembles the group of pursuit predation (Fig. 3a of the main manuscript), whereas group 2 (orange data points) and group 3 (blue data points) closely mimic the distributions of the data points for ambushing and group hunting, respectively.

Supplementary Figure 1: Clustering of the predator-prey links in three groups.

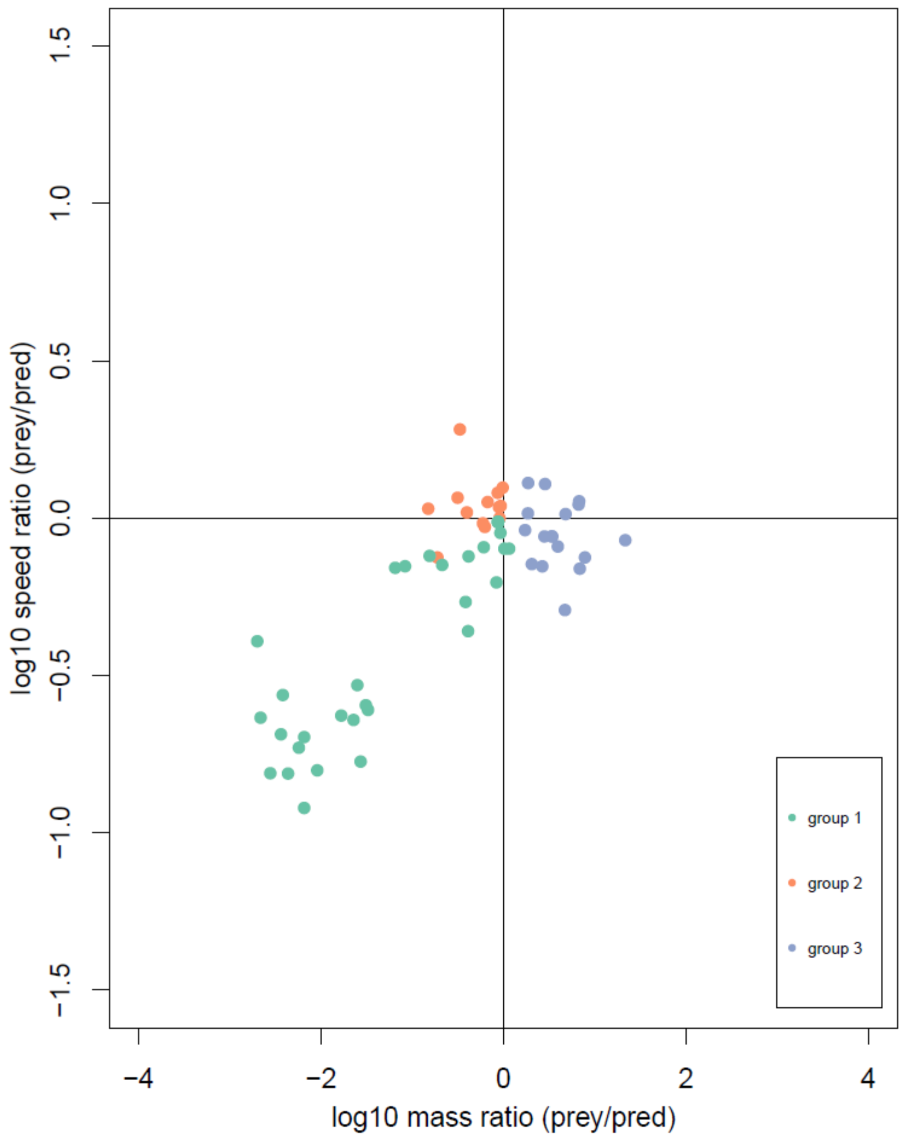

Supplementary Table 1 | Database on body masses and maximum speeds of mammalian predator-prey pairs.

predator species	predator mass [kg]	mass ref.	predator max. speed [km/h]	speed ref.	prey species	prey mass [kg]	mass ref.	prey max. speed [km/h]	speed ref.	link ref.	hunting strategy	comment
Acinonyx jubatus	65.00	1	120.00	1	*Aepyceros melampus*	54.42	1	74.99	1	6	pursuit predation	
Acinonyx jubatus	65.00	1	120.00	1	*Antidorcas marsupialis*	39.50	1	97.00	1	6	pursuit predation	
Acinonyx jubatus	65.00	1	120.00	1	*Eudorcas thomsonii*	25.00	1	65.00	1	6	pursuit predation	
Canis aureus	9.20	1	55.98	1	*Microtus spp.*	0.04	1	8.64	1	27	pursuit predation	
Canis aureus	9.20	1	55.98	1	*Mus musculus*	0.02	1	13.00	1	7	pursuit predation	
Canis aureus	9.20	1	55.98	1	*Rattus spp.*	0.25	1	9.43	1	7	pursuit predation	
Canis latrans	18.00	1	69.04	1	*Lepus americanus*	1.50	1	48.55	1	8	pursuit predation	
Canis latrans	18.00	1	69.04	1	*Microtus pennsylvanicus*	0.05	1	10.68	1	29	pursuit predation	
Canis latrans	18.00	1	69.04	1	*Odocoileus virgianus*	70.97	1	56.09	1	29	group hunting	
Canis latrans	18.00	1	69.04	1	*Spermophilus spp.*	0.30	1	16.28	1	8,30	pursuit predation	mean of all Spermophilus/Urocitellus species in (1)
Canis latrans	18.00	1	69.04	1	*Sylvilagus floridanus*	1.17	1	48.00	1	29	pursuit predation	
Canis lupus	40.00	1	63.97	1	*Bison bison*	865	1	54.43	1	14	group hunting	
Canis lupus	40.00	1	63.97	1	*Capreolus capreolus*	25	1	59.98	1	15	group hunting	
Canis lupus	40.00	1	63.97	1	*Cervus canadensis*	270	1	72.40	1	14	group hunting	
Canis lupus	40.00	1	63.97	1	*Sus scrofa*	135	1	56.00	1	15	group hunting	
Crocuta crocuta	63.00	3	62.32	1	*Aepyceros melampus*	54.42	1	74.99	1	9	pursuit predation	
Crocuta crocuta	63.00	3	62.32	1	*Connochaetes taurinus*	180.00	1	79.98	1	9	group hunting	
Crocuta crocuta	63.00	3	62.32	1	*Equus zebra*	302.03	1	64.19	1	9	group hunting	
Crocuta crocuta	63.00	3	62.32	1	*Eudorcas thomsonii*	25.00	1	65.00	1	9	pursuit predation	
Crocuta crocuta	63.00	3	62.32	1	*Oryx gazella*	37.50	3	60.00	37	9	pursuit predation	
Felis chaus	10.00	3	32.00	32	*Lepus capensis*	3.33	1	61.24	1	33	ambushing	mean of all Lepus species in (1)
Felis chaus	10.00	3	32.00	32	*Mus musculus*	0.02	1	13.00	1	33	pursuit predation	
Felis chaus	10.00	3	32.00	32	*Rattus spp.*	0.25	1	9.43	1	33	pursuit predation	
Leopardus pardalis	10.50	3	61.00	35	*Liomys pictus*	0.04	1	16.72	1	36	pursuit predation	
Leopardus pardalis	10.50	3	61.00	35	*Monodelphis spp.*	0.06	3	11.37	1	34	pursuit predation	mean mass of all Monodelphis species in (3) and speed data of M. brevicaudata
Lycaon pictus	27.00	1	72.42	1	*Aepyceros melampus*	50.00	1	74.99	1	16	group hunting	
Lycaon pictus	27.00	1	72.42	1	*Connochaetes taurinus*	180.00	1	79.98	1	16	group hunting	

Lycaon pictus	27.00	1	72.42	1	*Eudorcas thomsonii*	25.00	1	65.00	1	16	group hunting	
Lynx canadensis	9.68	3	64.00	5	*Lepus americanus*	1.50	1	48.55	1	10	pursuit predation	mean of all Lepus species in (1)
Lynx canadensis	9.68	3	64.00	5	*Spermophilus spp.*	0.30	1	16.28	1	10	pursuit predation	
Lynx canadensis	9.68	3	64.00	5	*Tamiasciurus hudsonicus*	0.22	1	14.62	1	10	pursuit predation	
Panthera leo	199.00	1	80.00	1	*Connochaetes gnou*	133.50	1	90.00	1	17	group hunting	
Panthera leo	199.00	1	80.00	1	*Connochaetes taurinus*	180.00	1	79.98	1	17	group hunting	
Panthera leo	199.00	1	80.00	1	*Giraffa camelopardalis*	1555.00	1	60.00	1	17	group hunting	
Panthera leo	199.00	1	80.00	1	*Oryx gazella*	37.50	3	60.00	37	17	group hunting	
Panthera leo	199.00	1	80.00	1	*Syncerus caffer*	529.77	1	56.23	1	17	group hunting	
Panthera leo	199.00	1	80.00	1	*Taurotragus derbianus*	680.77	1	69.98	1	17	group hunting	
Panthera leo	199.00	1	80.00	1	*Taurotragus oryx*	559.76	1	69.98	1	17	group hunting	
Panthera pardus	51.00	1	59.98	1	*Aepyceros melampus*	50.00	1	74.99	1	19	ambushing	
Panthera pardus	51.00	1	59.98	1	*Axis axis*	45.50	1	65.00	1	19	ambushing	
Panthera pardus	51.00	1	59.98	1	*Phacochoerus aethiopicus*	87.90	1	54.95	1	19	ambushing	
Panthera tigris	144.88	1	55.98	1	*Axis axis*	45.50	1	65.00	1	20	ambushing	
Panthera tigris	144.88	1	55.98	1	*Bos javanicus*	683.33	3	28.58	1	20	ambushing	speed data of Bos sauveli
Panthera tigris	144.88	1	55.98	1	*Capreolus capreolus*	21.67	3	59.98	1	20	ambushing	
Panthera tigris	144.88	1	55.98	1	*Cervus canadensis*	270	1	72.40	1	20	ambushing	
Panthera tigris	144.88	1	55.98	1	*Sus scrofa*	135.00	1	61.21	1	20	ambushing	
Panthera tigris	144.88	1	55.98	1	*Tapirus indicus*	296.25	3	39.99	1	20	ambushing	speed data of Tapirus terrestris
Puma concolor	53.70	3	80.00	2	*Alces americanus*	368.50	3	55.27	1	21	ambushing	speed data of Alces alces
Puma concolor	53.70	3	80.00	2	*Odocoileus hemionus*	54.95	1	63.97	1	21	ambushing	
Puma concolor	53.70	3	80.00	2	*Odocoileus virginianus*	61.94	1	63.97	1	21	ambushing	
Puma concolor	53.70	3	80.00	2	*Pecari tajacu*	21.98	1	34.99	1	22	ambushing	
Urocyon cinereoargenteus	5.50	1	67.60	1	*Microtus pennsylvanicus*	0.05	1	10.68	1	38	pursuit predation	
Urocyon cinereoargenteus	5.50	1	67.60	1	*Neotoma spp.*	0.18	3	16.62	1	11	pursuit predation	
Urocyon cinereoargenteus	5.50	1	67.60	1	*Peromyscus spp.*	0.02	1	13.90	1	11	pursuit predation	
Urocyon cinereoargenteus	5.50	1	67.60	1	*Sylvilagus floridanus*	1.17	1	48.00	1	11	pursuit predation	
Vulpes vulpes	4.59	1	72.00	1	*Antechinomys laniger*	0.03	1	14.51	1	13	pursuit predation	
Vulpes vulpes	4.59	1	72.00	1	*Antechinomys laniger*	0.03	1	14.51	1	13	pursuit predation	
Vulpes vulpes	4.59	1	72.00	1	*Lepus europaeus*	4.00	1	70.02	1	13	pursuit predation	
Vulpes vulpes	4.59	1	72.00	1	*Microtus oeconomicus*	0.03	3	8.64	1	13	pursuit predation	mean speed of M. pennsylvaticus and M. pinetorium
Vulpes vulpes	5.50	1	67.60	1	*Microtus pennsylvanicus*	0.05	1	10.68	1	38	pursuit predation	
Vulpes vulpes	5.5	1	67.60	1	*Neotoma spp.*	0.1804331	3	16.62	1	11	pursuit predation	mean mass of all Neotoma species in (3), speed of Neotoma lepida

Vulpes vulpes	4.59	1	72.00	1	*Oryctolagus cuniculus*	1.90	1	54.43	1	12	pursuit predation	
Vulpes vulpes	5.50	1	67.60	1	*Peromyscus spp.*	0.02	1	13.90	1	11	pursuit predation	mean mass and speed of all Peromyscus species in (1)

References

1 Hirt, M. R., W. Jetz, B. C. Rall, and U. Brose. 2017. A general scaling law reveals why the largest animals are not the fastest. Nature ecology & evolution 1:1116–1122.

2 http://mountainlion.org/FAQfrequentlyaskedquestions.asp, retrieved 04.08.2017

3 Myhrvold, N. P., E. Baldridge, B. Chan, D. Sivam, D. L. Freeman, and S. K. Ernest. 2015. An amniote life-history database to perform comparative analyses with birds, mammals, and reptiles. Ecology 96:3109–3109.

4 http://a-z-animals.com/animals/wolverine/, retrieved 24.11.2017, 15:56

5 http://dinoanimals.com/animals/the-fastest-animals-in-the-world-top-100/, retrieved 24.11.2017

6 Hayward, M. W., Hofmeyr, M., O'brien, J., & Kerley, G. I. H. (2006). Prey preferences of the cheetah (Acinonyx jubatus)(Felidae: Carnivora): morphological limitations or the need to capture rapidly

7 Jaeger, M. M., Haque, E., Sultana, P., & Bruggers, R. L. (2007). Daytime cover, diet and space-use of golden jackals (Canis aureus) in agro-ecosystems of Bangladesh. Mammalia, 71(1/2), 1–10.

8 Theberge, J. B., & Wedeles, C. H. R. (1989). Prey selection and habitat partitioning in sympatric coyote and red fox populations, southwest Yukon. *Canadian Journal of Zoology* , 67(5), 1285–1290.

9 Hayward, M. W. (2006). Prey preferences of the spotted hyaena (Crocuta crocuta) and degree of dietary overlap with the lion (Panthera leo). *Journal of Zoology* , *270* (4), 606–614.

10 Poole, K. G. (2003). A review of the Canada lynx, Lync canadensis, in Canada. *The Canadian Field- Naturalist* , *117* (3), 360–376.

11 Neale, J.C. & Sacks, B.N. (2001). Food habits and space use of gray foxes in relation to sympatric coyotes and bobcats. *Can. J. Zool.* , 79, 1794–1800

12 Catling, P. C. (1988). Similarities and contrasts in the diets of foxes, Vulpes vulpes, and cats, Felis catus, relative to fluctuating prey populations and drought. Wildlife Research, 15(3), 307–317.

13 Jędrzejewski, W., and B. Jędrzejewska. 1992. Foraging and diet of the red fox Vulpes vulpes in relation to variable food resources in Biatowieza National Park, Poland. Ecography 15:212–220.

14 Metz, M. C., Smith, D. W., Vucetich, J. A., Stahler, D. R., & Peterson, R. O. (2012). Seasonal patterns of predation for gray wolves in the multi-prey system of Yellowstone National Park. Journal of Animal Ecology, 81(3), 553–563.

15 Nowak, S., Mysłajek, R. W., Kłosińska, A., & Gabryś, G. (2011). Diet and prey selection of wolves (Canis lupus) recolonising Western and Central Poland. *Mammalian Biology* Zeitschrift Für Säugetierkunde, 76(6), 709–715.

16 Hayward, M. W., O'Brien, J., Hofmeyr, M., & Kerley, G. I. H. (2006). Prey preferences of the African wild dog *Lycaon pictus* (Canidae: Carnivora): ecological requirements for conservation. Journal of Mammalogy, 87(6), 1122–1131.

17 Hayward, M. W., & Kerley, G. I. H. (2005). Prey preferences of the lion (Panthera leo). *Journal of Zoology* , *267* (3), 309–322.

18 Myhre, R., & Myrberget, S. (1975). Diet of Wolverines (Gulo gulo) in Norway. *Journal of Mammalogy* , *56* (4), 752–757.

19 Hayward, M. W., Henschel, P., O'brien, J., Hofmeyr, M., Balme, G., & Kerley, G. I. H. (2006). Prey preferences of the leopard (Panthera pardus). *Journal of Zoology* , *270* (2), 298–313.

20 Hayward, M. W., Jędrzejewski, W., & Jędrzewska, B. (2012). Prey preferences of the tiger Panthera tigris. *Journal of Zoology* .

21 Iriarte, JA, Franklin, WL, Johnson, WE, Redford (1990), KH. Biogeographic variation of food habits and body size of the America puma. *Oecologia ,* 85, 185-190

22 Moreno, R. S., Kays, R. W., & Samudio Jr, R. (2006). Competitive release in diets of ocelot (Leopardus pardalis) and puma (Puma concolor) after jaguar (Panthera onca) decline. Journal of Mammalogy, 87(4), 808–816.

23 Holekamp, K. E., Smale, L., Berg, R., & Cooper, S. M. (1997). Hunting rates and hunting success in the spotted hyena (Crocuta crocuta). *Journal of Zoology* , *242* (1), 1–15.

24 Wilson, A. M., Hubel, T. Y., Wilshin, S. D., Lowe, J. C., Lorenc, M., Dewhirst, O. P., … Golabek, K. A. (2018). Biomechanics of predator–prey arms race in lion, zebra, cheetah and impala. Nature, 554(7691), 183.

25 Schaller, G. B. (1968). Hunting Behaviour of the Cheetah in the Serengeti National Park, Tanzania. *African Journal of Ecology* , *6* (1), 95–100.

26 Burger, J., Safina, C., & Gochfeld, M. (2000). Factors affecting vigilance in springbok: importance of vegetative cover, location in herd, and herd size. *Acta Ethologica* , *2* (2), 97–104.

27 Lanszki, J., Heltai, M., & Szabó, L. (2006). Feeding habits and trophic niche overlap between sympatric golden jackal (Canis aureus) and red fox (Vulpes vulpes) in the Pannonian ecoregion (Hungary). Canadian Journal of Zoology, 84(11), 1647–1656.

28 Janis, C. M., and B. Figueirido. 2014. Forelimb anatomy and the discrimination of the predatory behavior of carnivorous mammals: The thylacine as a case study. Journal of Morphology 275:1321–1338.

29 Cepek, J. D. (2004). Diet composition of coyotes in the Cuyahoga Valley National Park, Ohio. *The Ohio Journal of Science, 104 (3), 60-64.*

30 Wells, M. C., & Bekoff, M. (1982). Predation by Wild Coyotes: Behavioral and Ecological Analyses. *Journal of Mammalogy* , *63* (1), 118–127. doi: 10.2307/1380678

31 Thibault, I., & Ouellet, J.-P. (2005). Hunting behaviour of eastern coyotes in relation to vegetation cover, snow conditions, and hare distribution. *Écoscience* , *12* (4), 466–475.

32 Sunquist, M., & Sunquist, F. (2017). *Wild Cats of the World* . University of Chicago Press.

33 Mohammad, M. K. (2008). The Parasitic Fauna And The Food Habits Of The Wild Jungle Cat Felis chaus Furax De Winton, 1898 In Iraq. *Bull. Iraq Nat. Hist. Mus* , *10* (2), 65–78.

34 Wang, E. (2002). Diets of Ocelots (Leopardus pardalis), Margays (L. wiedii), and Oncillas (L. tigrinus) in the Atlantic Rainforest in Southeast Brazil. *Studies on Neotropical Fauna* and Environment, 37(3), 207–212.

35 https://a-z-animals.com/animals/ocelot/, retrieved 18.10.2019

36 Meyer, E. M., & GONZÁLEZ, C. A. L. (2002). Ocelot (Leopardus pardalis) food habits in a tropical deciduous forest of Jalisco, Mexico. *The American Midland Naturalist* , *148* (1), 146–155.

37 https://en.wikipedia.org/wiki/Gemsbok, retrieved 14.10.2019

38 Hockman, J. G., & Chapman, J. A. (1983). Comparative feeding habits of red foxes (Vulpes vulpes) and gray foxes (Urocyon cinereoargenteus) in Maryland. *American Midland* Naturalist, 276–285.

Supplementary Table 2 | Body-mass and maximum-speed ratios of the predator-prey pairs of Supplementary Table 1 given as ratios from predator to prey and prey to predator.

		predator/prey				prey/predator			
predator species	prey species	mass ratio	log10 (mass ratio)	speed ratio	log10 (speed ratio)	mass ratio	log10 (mass ratio)	speed ratio	log10 (speed ratio)
Acinonyx jubatus	*Aepyceros melampus*	1.19	0.08	1.60	0.20	0.84	-0.08	0.62	-0.20
Acinonyx jubatus	*Antidorcas marsupialis*	1.65	0.22	1.24	0.09	0.61	-0.22	0.81	-0.09
Acinonyx jubatus	*Eudorcas thomsonii*	2.60	0.41	1.85	0.27	0.38	-0.41	0.54	-0.27
Canis aureus	*Microtus spp.*	230.00	2.36	6.48	0.81	0.00	-2.36	0.15	-0.81
Canis aureus	*Mus musculus*	460.00	2.66	4.31	0.63	0.00	-2.66	0.23	-0.63
Canis aureus	*Rattus spp.*	36.80	1.57	5.94	0.77	0.03	-1.57	0.17	-0.77
Canis latrans	*Lepus americanus*	12.00	1.08	1.42	0.15	0.08	-1.08	0.70	-0.15
Canis latrans	*Microtus pennsylvanicus*	360.00	2.56	6.46	0.81	0.00	-2.56	0.15	-0.81
Canis latrans	*Odocoileus virgianus*	0.25	-0.60	1.23	0.09	3.94	0.60	0.81	-0.09
Canis latrans	*Spermophilus spp.*	60.00	1.78	4.24	0.63	0.02	-1.78	0.24	-0.63
Canis latrans	*Sylvilagus floridanus*	15.38	1.19	1.44	0.16	0.07	-1.19	0.70	-0.16
Canis lupus	*Bison bison*	0.05	-1.33	1.18	0.07	21.63	1.33	0.85	-0.07
Canis lupus	*Capreolus capreolus*	1.60	0.20	1.07	0.03	0.63	-0.20	0.94	-0.03
Canis lupus	*Cervus canadensis*	0.15	-0.83	0.88	-0.05	6.75	0.83	1.13	0.05
Canis lupus	*Sus scrofa*	0.30	-0.53	1.14	0.06	3.38	0.53	0.88	-0.06
Crocuta crocuta	*Aepyceros melampus*	1.16	0.06	0.83	-0.08	0.86	-0.06	1.20	0.08
Crocuta crocuta	*Connochaetes taurinus*	0.35	-0.46	0.78	-0.11	2.86	0.46	1.28	0.11
Crocuta crocuta	*Equus zebra*	0.21	-0.68	0.97	-0.01	4.79	0.68	1.03	0.01
Crocuta crocuta	*Eudorcas thomsonii*	2.52	0.40	0.96	-0.02	0.40	-0.40	1.04	0.02
Crocuta crocuta	*Oryx gazella*	1.68	0.23	1.04	0.02	0.60	-0.23	0.96	-0.02
Felis chaus	*Lepus capensis*	3.00	0.48	0.52	-0.28	0.33	-0.48	1.91	0.28
Felis chaus	*Mus musculus*	500.00	2.70	2.46	0.39	0.00	-2.70	0.41	-0.39
Felis chaus	*Rattus spp.*	40.00	1.60	3.39	0.53	0.03	-1.60	0.29	-0.53
Leopardus pardalis	*Liomys pictus*	262.50	2.42	3.65	0.56	0.00	-2.42	0.27	-0.56
Leopardus pardalis	*Monodelphis spp.*	175.00	2.24	5.36	0.73	0.01	-2.24	0.19	-0.73
Lycaon pictus	*Aepyceros melampus*	0.54	-0.27	0.97	-0.02	1.85	0.27	1.04	0.02
Lycaon pictus	*Connochaetes taurinus*	0.15	-0.82	0.91	-0.04	6.67	0.82	1.10	0.04
Lycaon pictus	*Eudorcas thomsonii*	1.08	0.03	1.11	0.05	0.93	-0.03	0.90	-0.05
Lynx canadensis	*Lepus americanus*	6.45	0.81	1.32	0.12	0.15	-0.81	0.76	-0.12
Lynx canadensis	*Spermophilus spp.*	32.27	1.51	3.93	0.59	0.03	-1.51	0.25	-0.59
Lynx canadensis	*Tamiasciurus hudsonicus*	44.00	1.64	4.38	0.64	0.02	-1.64	0.23	-0.64
Panthera leo	*Connochaetes gnou*	1.49	0.17	0.89	-0.05	0.67	-0.17	1.13	0.05
Panthera leo	*Connochaetes taurinus*	1.11	0.04	1.00	0.00	0.90	-0.04	1.00	0.00
Panthera leo	*Giraffa camelopardalis*	0.13	-0.89	1.33	0.12	7.81	0.89	0.75	-0.12
Panthera leo	*Oryx gazella*	5.31	0.72	1.33	0.12	0.19	-0.72	0.75	-0.12
Panthera leo	*Syncerus caffer*	0.38	-0.43	1.42	0.15	2.66	0.43	0.70	-0.15

Panthera leo	*Taurotragus derbianus*	0.29	-0.53	1.14	0.06	3.42	0.53	0.87	-0.06
Panthera leo	*Taurotragus oryx*	0.36	-0.45	1.14	0.06	2.81	0.45	0.87	-0.06
Panthera pardus	*Aepyceros melampus*	1.02	0.01	0.80	-0.10	0.98	-0.01	1.25	0.10
Panthera pardus	*Axis axis*	1.12	0.05	0.92	-0.03	0.89	-0.05	1.08	0.03
Panthera pardus	*Phacochoerus aethiopicus*	0.58	-0.24	1.09	0.04	1.72	0.24	0.92	-0.04
Panthera tigris	*Axis axis*	3.18	0.50	0.86	-0.06	0.31	-0.50	1.16	0.06
Panthera tigris	*Bos javanicus*	0.21	-0.67	1.96	0.29	4.72	0.67	0.51	-0.29
Panthera tigris	*Capreolus capreolus*	6.69	0.83	0.93	-0.03	0.15	-0.83	1.07	0.03
Panthera tigris	*Cervus canadensis*	0.54	-0.27	0.77	-0.11	1.86	0.27	1.29	0.11
Panthera tigris	*Sus scrofa*	1.07	0.03	0.91	-0.04	0.93	-0.03	1.09	0.04
Panthera tigris	*Tapirus indicus*	0.49	-0.31	1.40	0.15	2.04	0.31	0.71	-0.15
Puma concolor	*Alces americanus*	0.15	-0.84	1.45	0.16	6.86	0.84	0.69	-0.16
Puma concolor	*Odocoileus hemionus*	0.98	-0.01	1.25	0.10	1.02	0.01	0.80	-0.10
Puma concolor	*Odocoileus virginianus*	0.87	-0.06	1.25	0.10	1.15	0.06	0.80	-0.10
Puma concolor	*Pecari tajacu*	2.44	0.39	2.29	0.36	0.41	-0.39	0.44	-0.36
Urocyon cinereoargenteus	*Microtus pennsylvanicus*	110.00	2.04	6.33	0.80	0.01	-2.04	0.16	-0.80
Urocyon cinereoargenteus	*Neotoma spp.*	30.56	1.49	4.07	0.61	0.03	-1.49	0.25	-0.61
Urocyon cinereoargenteus	*Peromyscus spp.*	275.00	2.44	4.86	0.69	0.00	-2.44	0.21	-0.69
Urocyon cinereoargenteus	*Sylvilagus floridanus*	4.70	0.67	1.41	0.15	0.21	-0.67	0.71	-0.15
Vulpes vulpes	*Antechinomys laniger*	153.00	2.18	4.96	0.70	0.01	-2.18	0.20	-0.70
Vulpes vulpes	*Antechinomys laniger*	153.00	2.18	4.96	0.70	0.01	-2.18	0.20	-0.70
Vulpes vulpes	*Lepus europaeus*	1.15	0.06	1.03	0.01	0.87	-0.06	0.97	-0.01
Vulpes vulpes	*Microtus oeconomicus*	153.00	2.18	8.33	0.92	0.01	-2.18	0.12	-0.92
Vulpes vulpes	*Microtus pennsylvanicus*	110.00	2.04	6.33	0.80	0.01	-2.04	0.16	-0.80
Vulpes vulpes	*Neotoma spp.*	30.48	1.48	4.07	0.61	0.03	-1.48	0.25	-0.61
Vulpes vulpes	*Oryctolagus cuniculus*	2.42	0.38	1.32	0.12	0.41	-0.38	0.76	-0.12
Vulpes vulpes	*Peromyscus spp.*	275.00	2.44	4.86	0.69	0.00	-2.44	0.21	-0.69
